# Sunk Cost Effects for Time Versus Money: Replication and Extensions Registered Report of Soman (2001)

**DOI:** 10.5334/irsp.883

**Published:** 2023-11-28

**Authors:** Nikolay B. Petrov, Yin Kan Megan Chan, Cheuk Nam Lau, Tin Ho Kwok, Lok Ching Estelle Chow, Wai Yan Lo, Wenkai Song, Gilad Feldman

**Affiliations:** 1 University of Cambridge, UK; 2 Students at the Department of Psychology, University of Hong Kong during the academic year 2021/22, HK; 3 Department of Psychology, University of Hong Kong, Hong Kong SAR, HK; 4 School of Entrepreneur and Management, ShanghaiTech University, Shanghai, CN; 5 Department of Psychology, University of Hong Kong, HK

**Keywords:** judgment and decision-making, sunk cost, escalation of commitment, time, money, opportunity cost

## Abstract

The sunk cost effect is the tendency for an individual’s decision making to be impacted by unrecoverable previous investments of resources. Soman (2001) found that sunk cost effect is weaker for time than for money (Studies 1 and 2) and that the facilitation of money-like accounting strengthens the sunk cost effect for time (Study 5). We conducted a Registered Report of a close, high-powered replication and extension of Soman’s (2001) Studies 1 and 2 and a conceptual replication of his Study 5 with an online sample of US American Amazon Mechanical Turk (*N* = 821). We found support for differences between sunk money costs and sunk time costs in Study 1 (original: ϕ_c_ = .61 [.43, .78]; replication: ϕ_c_ = .38 [.31, .45]), yet not in Study 2, in which we found sunk cost effects for both money and time (original: money – ϕ_c_ = .32 [.12, .52], time – ϕ_c_ = .02 [.00, .18]; replication: money – ϕ_c_ = .23 [.14, .33], time – ϕ_c_ = .32 [.23, .42]). In Study 5, we found no support for facilitation of money-like accounting as strengthening the sunk time cost effect. Materials, data, and code are available on: https://osf.io/pm264/.

## PCIRR-Study Design Table

**Table:** 


QUESTION	HYPOTHESIS	SAMPLING PLAN	ANALYSIS PLAN	RATIONALE FOR TEST	INTERPRETATION GIVEN DIFFERENT OUTCOMES	THEORY THAT COULD BE SHOWN WRONG BY THE OUTCOMES

Is the sunk cost effect weaker for time than for money?	The sunk cost effect is weaker for time than for money.	Participants recruited online using the US American Amazon platform.	Chi-square test	We follow the statistical methods of the original paper.	Based on the criteria used by LeBel et al. (2018) we will examine the replicability of the findings of Soman (2001).	The sunk cost effect is weaker for time than for money and the facilitation of money-like accounting for sunk time costs strengthens the sunk cost effect.
	
Does the facilitation of money-like accounting for sunk time costs strengthen the sunk time cost effect?	Facilitation of money-like accounting by using education about economic approaches to time strengthens the sunk cost effect of time		Two-way between-subject ANOVA			


People will often increase time and money investments in a failing course of action to try and recover or justify an initial investment, leading to an escalating commitment to a losing course of action. This phenomenon has been coined the ‘sunk cost effect’ (Arkes & Blumer, 1985; Thaler, 1980), given that with larger sunk costs there are stronger tendencies to further escalate.

The sunk cost effect has mostly been investigated with the invested resources being either money or time (or both, e.g., Pandey & Sharma, 2019). In the money domain, findings have been largely consistent and in support of sunk money effects (Arkes & Blumer, 1985; Bornstein et al., 1999; Coleman, 2009; Navarro & Fantino, 2005; Soman & Cheema, 2001), though there were several failures, such as that of Friedman et al. (2007).

Compared to sunk money costs, sunk time costs seem more volatile. For example, Navarro and Fantino (2009) found that undergraduate students were susceptible to sunk time effects across various factors, including the difficulty of and enjoyment from the future time investment and personal responsibility. Silva Castillo et al. (2020) also found evidence for sunk time costs in a within-subject study of 46 undergraduate students, also showing that there is a positive linear relationship between the time investment and the subjective value placed on the outcome. Bornstein and Chapman (1995) similarly found evidence of sunk time costs, with the presence and strength of these differences being affected by other factors, such as who the decision maker is in the scenario and how carefully the decision is considered. In comparing money and time for sunk costs, Park and Jang (2014) found that among people from the general population both sunk time and sunk money costs independently predicted intentions to cancel a future hypothetical trip. In a similar vein, Pandey and Sharma (2019), across three vignette experiments, found that found that graduate students were susceptible to sunk time costs both in scenarios when they spend only time and when they spend money and time both, but money can be recovered. However, in this study, the sunk time cost effect only appeared when the time investment exceeded a specific threshold, which raises the question of what other factors affect the different expressions of the sunk money and sunk time effects.

Some research already points to potential candidates that distinguish between sunk money and sunk time effects. For instance, across online and field studies, Soster et al. (2010) showed that the sunk money and sunk time effects are equivalent if the accounting period is the same, but asymmetrical if the accounting periods are different. Another example comes from Okada and Hoch (2004), who showed that both risk aversion and ambiguity in the outcome produce differences in how time and money costs are accounted for.

Another factor that might differentially affect sunk money and sunk time costs is age. Strough et al. (2008) showed that younger adults are less likely to be susceptible to sunk money costs. One way to contextualise this finding is to consider that sunk costs are not taken in their absolute values, but relative to an individual’s total available resource (Garland & Newport, 1991), and older adults are generally wealthier and have less available time, compared to younger adults. Another contextualising factor for the age effect is that experience accounting for both time and money has been shown to predict susceptibility to sunk costs, and younger people likely have much less experience (Bornstein et al., 1999; DeVoe & Pfeffer, 2007; Ronayne et al., 2021).

Methods-wise, Rego et al. (2018) showed that although participants are more likely to stay in an unhealthy relationship when money, but not time, was invested, the effect of sunk time costs was stronger when the outcome was measured on a continuum scale (amount of time willing to invest in an unhappy relationship) rather than as a binary choice (whether or not to invest time).

Overall, although these studies hint at some factors that might affect sunk money and sunk time effects differently, the underlying reasons for these differences remain unclear.

To address this question, Soman (2001) focused on three reasons that make accounting for the sunk costs of time more difficult compared to those of money: 1) time cannot be inventoried or replaced, 2) time is not as easily aggregated as money, 3) accounting for money, unlike time, is a routine activity. In this seminal work, Soman (2001) asked participants, across several experiments, to read scenarios that only differed in whether they were related to time or money and whether there were any sunk costs to be accounted for. Soman’s (2001) core finding was that the strength of the sunk cost effect was weaker for time than for money. He further showed that the facilitation of money-like accounting for sunk time costs by highlighting opportunity costs or by educating about an economic approach to time strengthens the sunk time cost effect.

The ubiquity of sunk costs in everyday life and the impact of Soman’s (2001) work (680 citations on Google Scholar as of November 2023) suggests the value of revisiting and expanding on this work. To the best of our knowledge, Soman’s (2001) research has not been directly replicated.

We aimed to revisit the classic phenomenon and examine the reproducibility and replicability of the classic findings by replicating the studies and improving the design with extensions. Following the recent growing recognition of reproducibility and replicability in psychological science (Brandt et al., 2014; Open Science Collaboration, 2015; Nosek et al., 2022; Zwaan et al., 2018), we embarked on a well-powered pre-registered replication and extensions of Soman (2001).

We focused our replication on Studies 1 and 2 as they provided the baseline test of the core hypothesis to elucidate whether people account for both the magnitude (Study 1) and the presence (Study 2) of sunk costs in each domain. We also targeted Study 5 in a conceptual replication as it suggested a method for potential mitigation of the effect. We summarized the hypotheses and effects for Studies 1, 2, and 5 in Table 1.

**Table 1:** Soman (2001): Summary of studies and hypotheses and a comparison of original and replication effects.


HYPOTHESES	STUDY	DESCRIPTION	STATISTICAL TEST	ORIGINAL OR REPLICATION	EFFECT SIZE^a^ [95% CI]	REPLICATION OUTCOME^b^

**Hypothesis 1:** The sunk-cost effect is weaker in the domain of temporal costs than in the domain of monetary costs.	**1 (Theatre and concert tickets)**	Two types of tickets are expressed in two different types of sunk cost domains—either time or money to investigate the relative strength of each domain.	Chi-square; difference between sunk time and sunk money conditions in rate of choosing a ticket	Original	ϕ_c_ = .61 [.43, .78]	signal – inconsistent, smaller

				Replication	ϕ_c_ = .38 [.31, .45]	

	**2 (Choosing a project)**	The domain (time/money) and the existence of sunk cost (present/absent) are manipulated within a scenario, describing potential projects to work on to test the strength of the sunk cost effects across domains.	Chi-square; difference between sunk time and no sunk time conditions in rate of choosing a project	Original	ϕ_c_ = .02 [.00, .18]	signal – inconsistent, positive

				Replication	ϕ_c_ = .32 [.23, .42]	

			Chi-square; difference between sunk money and no sunk money conditions in rate of choosing a project	Original	ϕ_c_ = .32 [.12, .52]	signal – consistent

				Replication	ϕ_c_ = .23 [.14, .33]	

**Hypothesis 2a:** If the absence of a sunk time cost effect is due to difficulties associated with the accounting of time, then the facilitation of accounting should cause the effect to reappear. [Alternative hypothesis] **Hypothesis 2b:** If the absence of a sunk time cost effect is due to the fact that individuals behave rationally when evaluating past time investments, then the facilitation of accounting should not cause the effect to reappear. [Null hypothesis]	**5 (Education and opportunity costs)**	The level of opportunity cost (high/low) and education (present/absent) were manipulated to evaluate the strength of sunk cost effects.	ANOVA; opportunity cost main effect	Original	= .09 [.00, .23]	no signal – inconsistent

				Replication	= .00 [.00, .01]	

			ANOVA; education main effect	Original	= .17 [.04, .32]	no signal – inconsistent

				Replication	= .00 [.00, .01]	

			ANOVA; opportunity cost by education interaction	Original	= .00 [.00, .02]	no signal – consistent

				Replication	= .00 [.00, .01]	


^a^ We provide additional detail regarding the calculation of effect sizes in the supplementary materials “Effect sizes calculation”. ^b^ We classified each effect using the criteria set out by LeBel et al. (2019).

## Studies Overview: Replications of Studies 1, 2, and 5

### Open Science Declaration

This project was submitted as a Registered Report (Chambers & Tzavella, 2022; Nosek & Lakens, 2014; Scheel et al., 2021; Wiseman et al., 2019), and received Peer Community in Registered Report Stage 1 in-principle acceptance (https://osf.io/65htv/; https://rr.peercommunityin.org/articles/rec?id=187) after which we created a frozen pre-registration version of the entire Stage 1 packet (https://osf.io/78vgx/) and proceeded to data collection. We provided all materials, data, and code on: https://osf.io/pm264/.

All measures, manipulations, and exclusions conducted for this investigation are reported, and data collection was completed before analyses.

We reported results after exclusions below, and in the supplementary materials, we detailed a comparison between pre- and post-exclusion findings as well as any deviations from the pre-registered plan (‘Comparisons and deviations’ subsection), with additional disclosures (‘Open science disclosures’ subsection).

### Procedure

We focused on Soman’s (2001) Studies 1, 2, and 5. We combined the three studies into a unified single data collection. This allowed us to maximize our resources and had the added advantage that we can rule out any sample characteristics that might be driving differences in successful versus unsuccessful replications. Additionally, a single unified survey allowed us to conduct additional exploratory within-subjects analyses and explore links between different studies, something that is not possible with the design of the original. Given that the replication of Study 5 involved education about sunk time costs with a scenario that was first introduced in Study 1, we fixed the order so that Study 5 is always last, with randomized order for the replications of Studies 1 and 2.

Participants first provided consent, after which they read an outline for the studies and three questions confirmed participants qualifications as being American, their understanding of the study procedures, and their agreement to pay close attention (Yes/No/Not sure presented in random order, and participants not answering Yes were asked to return the task). Participants then completed three studies: first Studies 1 and 2 in randomized order, followed by Study 5. In each of the studies, participants read a hypothetical scenario presenting them with two alternatives. In all studies, participants indicated their choice between the two alternatives, and in Studies 1 and 5 they also indicated their preference between the two options on a Likert scale (see below). After Studies 1 and 2, they were asked comprehension checks question to check if they understood the critical information in the scenario and afterwards asked if they had seen the scenario before, and if so, where. After completing all studies, participants answered questions inquiring about their seriousness and familiarity with the materials, reported their experience during the survey, and provided demographic information (with no implications for participation or pay). Finally, participants were thanked and debriefed. Throughout the study, participants could not go back to previous pages. Our replication project received ethical approval from the University of Hong Kong (REF ID: EA220438).

A methodological comparison between the original and the current study on key dimensions can be found in Table 2.

**Table 2:** Original versus replication methodological comparison.


	ORIGINAL	REPLICATION	REASON FOR CHANGE

Participants	Undergraduate students from Hong Kong University of Science and Technology and University of Colorado.	Participants from CloudResearch/Amazon MTurk.	Larger more diverse sample. Addressing sample concerns and allowing for exploratory analyses comparing effects across studies.

	Study 1, 2 and 5 were done separately with different participants.	Study 1, 2 and 5 were done in the same survey with the same participant.	

Delivery	Paper questionnaires	Online questionnaire using Qualtrics	

Questions	The original studies did not use any comprehension checks or instructional manipulation checks.	We used comprehension and instructional manipulation checks in our replication.	To ensure that participants read and understood the materials.

Materials	In Study 5, a class on opportunity cost was delivered to those in the education condition.	A passage about opportunity cost along with questions about that passage as instructional manipulation checks were presented.	To adjust to an online sample, we used a passage that participants read instead of a class.

Scale	In Study 5 the preference scale was originally from 1 to 9 and presented as such.	We adjusted the presentation of the scale to 4/0/4 instead of 1 to 9.	Avoid biasing participants in a certain direction.

Order of studies	Study 1 –> Study 2 –> Study 5	Randomized the order of studies 1 and 2 only, but not study 5. Study 5 is presented last at the end of the experiment.	To address potential impact of presentation order.


*Note*. Effect size for Study 1 and 2 is ϕ_c_ and for Study 5 – ; see “Effect sizes calculation” section in the supplementary materials.

### Materials

The descriptions of the stimuli in the target article were limited. We reached out to the author and received the materials used in the original, and we are very grateful for the author’s support in making these available. We used the same content with the minor exception that we started each scenario with ‘Imagine you are a student’ to adjust to the different sample (undergraduates vs. general population, see Table 2). We made some minor stylistic changes to the presentation of the materials (using bold/underline/italics at places). The survey used is available on the OSF, and a summary of the materials and questions used is provided in the supplementary materials (‘Materials used’ subsection).

### Power analysis

We used a ‘small-telescope’ approach in planning our sample size (Simonsohn, 2015). This approach allows us to both achieve the power to reject a zero-effect null hypothesis, assuming there is a true effect, and to detect an effect much smaller than the original could have possibly detected. To achieve this, it is recommended to use a replication sample 2.5 times that of the original. This is an especially powerful approach in conjunction with our implementation of the studies by combining them into a single survey as it means that powering the largest study entails giving even more power to the other ones. Thus, given that Soman (2001) used a sample size of 206 in his Study 2, we calculated a needed sample of at least 515 participants. However, we also wanted to test whether the order in which the studies was presented (Study 1 first vs. Study 2 first) affected the results, thus we doubled that sample and planned for a 15% planned exclusion rate, meaning we aimed to recruit 1212 participants in order to get a total of 1030 participants, with equal numbers completing Study 1 or Study 2 first.

We conducted a sensitivity analysis for both the 515 and 1030 target samples. We found that we had 99%+ power to detect the original smallest original effect sizes in each study and 80% power to detect effect sizes at least half of those of the original—see Table 3.

**Table 3:** Power analysis.


STUDY	SMALLEST EFFECT SIZE FROM ORIGINAL	POWER TO DETECT SMALLEST EFFECT SIZE FROM ORIGINAL		SMALLEST EFFECT SIZE DETECTABLE WITH 80% POWER	
	
		*N* = 515	*N* = 1030	*N* = 515	*N* = 1030

Study 1	.61	99%+	99%+	.12	.09

Study 2	.32	99%+	99%+	.17	.12

Study 5	.31	99%+	99%+	.13	.09


*Note*. Effect size for Study 1 and 2 is ϕ_c_ and for Study 5 – ; see “Effect sizes calculation” section in the supplementary materials.

### Exclusion criteria

We excluded participants who indicated low proficiency in English and the understanding of our materials (<5 on a 1–7 scale), low seriousness (<4 on 1–5 scale), familiarity with the materials (answered ‘Yes’ to seeing these materials before either at the end or at any of the two familiarity checks in Study 1 and 2), failure to comprehend the scenarios (inaccurate response on a) a question whether the scenario was about time or money and b) a question about whether their understanding of the materials was accurate, after ensuring they have understood the critical information), and participants who dropped out and failed to complete all three studies. We report the number of people excluded for each criterion and analyze their effect in the ‘Pre-exclusion versus post-exclusion results comparison’ section in the supplementary materials. Overall, we found no differences in conclusions when comparing analyses run on participants before exclusions and after exclusions.

### Participants

Overall, 1348 participants started the survey with 821 participants passing all exclusion criteria^1^ and were included in the final analyses (*M*_age_ = 44.03, *SD*_age_ = 12.79; 52.01% males, 47.02% females). We provide details of the final sample and a comparison to Soman’s (2001) samples in Table 4.

**Table 4:** Comparison of the Soman’s (2001) and the current sample.


	SOMAN (2001)	REPLICATION

Sample size	Study 1: 122 Study 2: 206 Study 5: 72	821

Geographic origin	Study 1: Hong Kong Study 2: US American Study 5: US American	US American Amazon Mechanical Turk workers

Gender	Undisclosed	427 males, 386 females, 8 other/did not disclose

Median age (years)	Undisclosed	42

Average age (years)	Undisclosed	44.03

Standard deviation age (years)	Undisclosed	12.79

Age range (years)	Undisclosed	20-82

Medium (location)	Study 1: Physical survey Study 2: Physical survey Study 5: Physical survey	Computer (online)

Compensation	Study 1: Credit Study 2: Undisclosed Study 5: Undisclosed	Nominal payment

Year	2001	2023

Sample source	Undergraduate students	General population


We recruited native English speakers who were born, raised, and located in the US on Amazon Mechanical Turk using the CloudResearch/TurkPrime platform (Litman et al., 2017). Based on our extensive experience of running similar judgment and decision-making replications on MTurk, to ensure high-quality data collection, we employed the following CloudResearch options: Duplicate IP Block. Duplicate Geocode Block, Suspicious Geocode Block, Verify Worker Country Location, Enhanced Privacy, CloudResearch Approved Participants, Block Low Quality Participants, and the like. We also employed Qualtrics’ fraud and spam prevention measures: reCAPTCHA, prevent multiple submissions, prevent ballot stuffing, bot detection, security scan monitor, and relevantID. We provide more details in the ‘Additional information about the study’ subsection in the supplementary materials.

The assignment pay was calculated based on the federal wage of 7.25USD/hour (though we did not restrict participation based on state-level minimum wage). We first pretested survey duration with 30 participants to make sure our time run estimate was accurate and then adjust pay as needed. The data from the 30 participants was not analyzed separately from the rest of the sample other than to assess survey completion duration and needed pay adjustments. For those pretest participants, if the survey duration was longer than expected, they were paid a bonus as a pay adjustment. Additionally, we used the feedback given by those participants to improve the quality of the survey. Specifically, we found typos in our original implementation, which we fixed, and identified an inconsistency in one of the conditions in Study 1, which we addressed after consulting with the author of the target article (see ‘Method’ of ‘Replication of Study 1’ below). We also added a brief note at the beginning to let participants know that failure to pass comprehension and attention checks or answer in a certain way will not result in rejections so they should answer to the best of their ability.

## Replication of Study 1

Study 1 was meant to test the first hypothesis that the sunk cost effect is weaker for time than for money. Participants read a hypothetical scenario about having invested either time or money and needed to decide whether to invest further resources into a preferred or a non-preferred option. We provided more information on the stimuli, procedure and measures in the supplementary materials (“Materials used” subsection).

### Method

#### Design and procedure

We employed a between-subject design with random allocation in either time or money sunk cost condition. In both conditions, the dependent variables were the same: a two-alternative forced choice, like the original, and a continuous preference scale, which we added (see next section).

Both the sunk time and the sunk money conditions asked participants to imagine that they had invested more resource (time or money)^2^ for a ticket for a theatre performance compared to the resource (time or money) invested for a ticket for a rock concert, but that they preferred going to the rock concert.

### Measures

#### Two-alternative forced choice (replication)

Participants then had to decide whether they would prefer to go to the theatre performance or the rock concert.

#### Preference (extension)

Because Study 5 employed very similar scenarios, we wanted to compare the responses from Study 1 to those of Study 5. To do so, we added in Study 1 the same measure as the original Study 5, which asked participants to indicate their preference on a scale of 1 (rock concert) to 9 (theatre performance). The scale was presented to participants as 4 (*Definitely Rock Concert*) through 0 (*Indifferent*) to 4 (*Definitely Theatre Performance*). A higher score on the scale represents less susceptibility to sunk cost fallacy. As a preliminary insight, in our replication of Study 5, we added the two-alternative forced choice that the current Study 1 had with the same aim of comparing responses across studies.

### Results

#### Two-alternative forced choice (replication)

**Figure 1:**
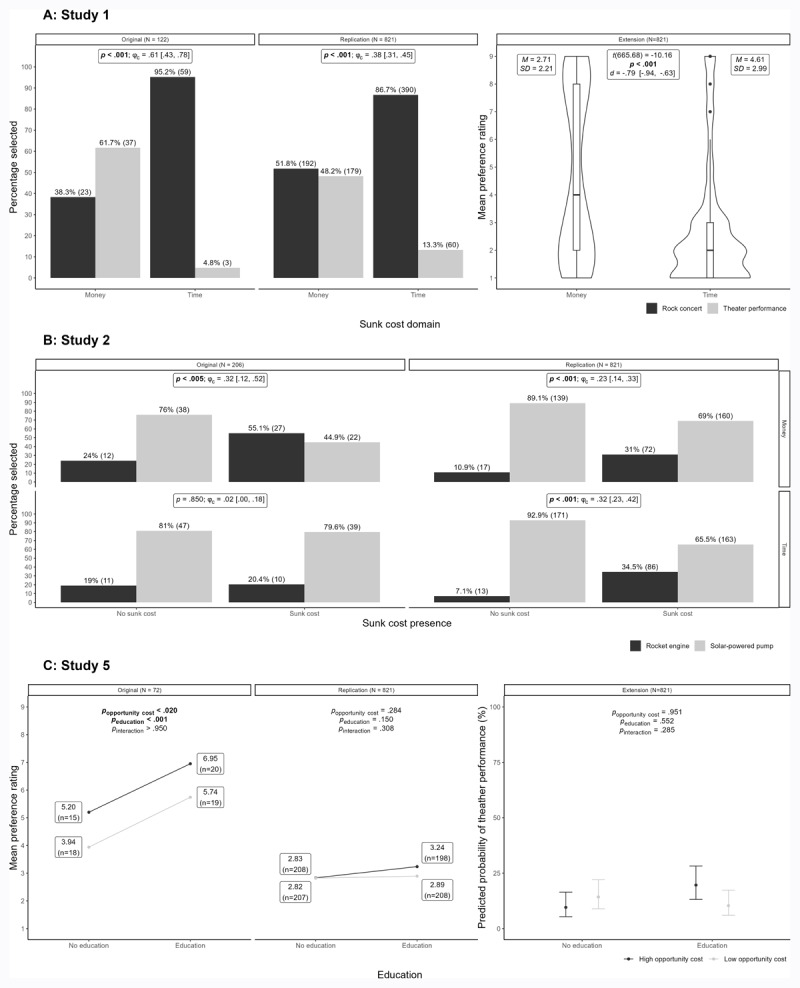
Summary of results comparing Soman’s original studies to the current replication effort. *Note*. Bold indicates statistically significant correlations.

We conducted a chi-square test and found support for differences in participants’ choice of theatre performance versus rock concert ticket between the sunk time cost (13.3% chose theatre performance ticket) and sunk money cost (48.2% chose theatre performance ticket) conditions, χ^2^(1) = 120.12, *p* < .001, ϕ_c_ = .38, 95% CI [.31, .45] – see Figure 1A).

Similarly, the original study found that 4.8% of participants preferred the theatre performance ticket in the sunk time condition, and 61.7% in the sunk money condition, thereby also showing a strong effect of sunk cost domain, χ^2^(1) = 44.68, *p* < .001, ϕ_c_ = .61 95% CI [.43, .78].

#### Preference (extension)

We conducted an independent samples t-test and found support for differences between the preference ratings of people in the time condition (*M* = 2.71, *SD* = 2.21) compared to those in the money condition (*M* = 4.61, *SD* = 2.99), *t* (665.68) = –10.16, *p* < .001, *d* = –.79 [–.94, –.63] (see Figure 1A).

### Discussion

In our replication of the target’s Study 1, we found support for differences between sunk money and sunk time, albeit with weaker effects than that of the target article, that the probability of the sunk cost effect is greater for money than for time, thereby supporting H1.

## Replication of Study 2

In this study, we further interrogated the first hypothesis, namely that the sunk cost effect is weaker for time than for money, by building on the previous study by adding another condition: whether there is a sunk cost or not. This allowed us to test whether the sunk cost effect would appear when comparing sunk cost versus no sunk cost conditions in each domain (time/money).

### Method

#### Design

We employed a 2 (*sunk cost domain*: time or money) × 2 (*sunk cost presence*: sunk cost or no sunk cost) between-subjects design with random allocation. In all conditions, the dependent variable was the same two-alternative forced choice.

#### Procedure

In the sunk cost conditions (regardless of the sunk cost domain), participants were asked to imagine that they had already invested substantial resources in developing a new rocket engine invention for a competition compared to no resource invested in developing a solar-powered pump. To finish either project would require the same resources, but they learn that the winner of last year’s competition also worked on a rocket engine design. They are then asked whether they would prefer to continue working on the rocket engine design (on which they have already spent resources) or to complete a solar-powered pump design.

In the no sunk cost condition, participants are presented with the same story, but they are not told that they had already invested resources in either design. We provided additional details in the ‘Materials used’ subsection of the supplementary materials.

### Results

We conducted two chi-square tests to analyze the difference between the sunk cost and no sunk cost conditions in each domain (time and money).

With time sunk cost, we found support for differences between those that read the sunk cost scenario (34.5% chose the rocket engine) and those who read the no sunk cost scenario (7.1% chose the rocket engine) in choosing which design to work on, χ^2^(1) = 45.28, *p* < .001, ϕ_c_ = .32, 95% CI [.23, .42].

With money sunk cost, we also found support for differences between those that read the sunk cost scenario (31.0% chose the rocket engine) and those who read the no sunk cost scenario (10.9% chose the rocket engine) in choosing which design to work on, χ^2^(1) = 21.40, *p* < .001, ϕ_c_ = .23, 95% CI [.14, .33] – see Figure 1B).

In comparison, the original study also found support for an effect in the money domain (55.1% chose the rocket engine in the sunk cost, and 24.0% in the no sunk cost, χ^2^(1) = 10.03, *p* = .002, ϕ_c_ = .32, 95% CI [.12, .52]), yet, unlike in our replication, found no effect in the time domain (20.4% chose the rocket engine in the sunk cost, and 19.0% in the no sunk cost, χ^2^(1) = .04, *p* = .852, ϕ_c_ = .02, 95% CI [.00, .18])

In summary, we found support for differences between sunk cost and no sunk cost conditions in both time and money domains. This conclusion is in line with the original’s findings for the money domain, albeit with a smaller effect size, but not in line with the findings for the time domain, which we found comparable to larger effects compared to the money domain.

### Discussion

In our replication we found support for sunk cost effect in the money domain, although weaker than that in the target article. However, unlike in the target article, we also found support for sunk costs effect in the time domain. Crucially, the results of the target’s Study 2 indicate that the sunk cost effect in the time domain were greater than in the money domain, thereby not in line with H1, not in line with the target’s Study 2 results, and diverging from the support for H1 that we found in our replication of Study 1.

## Replication of Study 5

In this study, we tested the second hypothesis, namely whether the facilitation of accounting for time strengthens the sunk time cost effect. To do this, we presented participants with a few paragraphs aimed at educating them about economic approaches to time. Additionally, we also varied the magnitude of the opportunity cost, such that it could be either low or high. This setup allowed us to test not only whether the education intervention works, but also the conditions in which that occurs.

### Method

#### Design

We employed a 2 (*opportunity cost*: low or high) × 2 (*education*: education or no education) between-subjects design with random allocation. In all conditions, the dependent variables were the same: a continuous preference scale, like the original, and a two-alternative forced choice, which we added (see next section).

#### Procedure

The scenario was similar to the one used in the replication of Study 1 with two differences. First, in the high opportunity cost condition, participants were told that they were ‘badly pressed for time,’ while in the low opportunity cost condition they were told that there is ‘relative flexibility in your schedule.’ Second, an education intervention was implemented: those who received education about opportunity costs were asked to read a short passage, which explained what an opportunity cost is and gave a thorough example. We provided additional details in the ‘Materials used’ subsection of the supplementary materials.

#### Measures

##### Preference (replication)

Participants indicated their preferences on a scale of 1 (*Rock Concert*) to 9 (*Theatre Performance*) which we presented to participants as 4 (*Definitely Rock Concert*), 0 (*Indifferent*) and 4 (*Definitely Theatre Performance*). A higher score on the scale represents less susceptibility to sunk cost fallacy. We note that this is a deviation from the original’s measure that ranged from 1 to 9 in presentation. We made this adjustment to avoid biasing participants towards the option presented with larger numbers.

##### Forced choice (extension)

To be able to compare the findings of Study 1 with that of Study 5 that employed similar stimuli we added the same two-alternative forced choice measure that was used in Study 1. As in Study 1 (above), participants had to decide whether they would prefer to go to the theatre performance or the rock concert.

### Results

#### Preference (replication)

To analyze the effects of opportunity cost and education on preference ratings for one ticket or the other, we selected Type-III ANOVA (to account for any variance in potential interactions; Field 2017). Assumptions for normality, outliers and homogeneity of variances were met, although ANOVA is robust to these violations with large samples (Blanca et al., 2017).

We conducted a 2 (Opportunity cost) × 2 (Education) between-groups ANOVA on preference ratings. We found no support for a main effect of opportunity cost, *F*(1, 817) = 1.15, *p* = .284, *ω^2^* = .00, η2*p* = .00, 95% CI [.00, .01] with those in the high opportunity cost condition (*M* = 3.03, *SD* = 2.43) not providing statistically different preference ratings than those in the low opportunity cost condition (*M* = 2.86, *SD* = 2.24). We also found no support for a main effect of education, *F*(1, 817) = 2.08, *p* = .150, *ω^2^* = .00, η2*p* = .00, 95% CI [.00, .01] with no support for differences in preference ratings between those in the education condition (*M* = 3.06, *SD* = 2.46) and those in the no-education condition (*M* = 2.83, *SD* = 2.21). We found no support for an interaction effect between the independent variables (*F* = 1.04, *p* = .308 – see Figure 1C).

In comparison, in the original study they found a main effect of opportunity cost, *F*(1, 68) = 6.63, *p* < .020, *ω^2^* = .073, η2*p* = .089, 95% CI [.00, .23], with those in the high opportunity cost condition (*M* = 6.20)^3^ providing higher preference ratings that those in the low opportunity cost condition (*M* = 4.86). The original study also found a main effect of education, *F*(1, 68) = 13.65, *p* < .001, *ω^2^* = .149, η2*p* = .167, 95% CI [.04, .32], with those who underwent education (*M* = 6.36) providing higher preference ratings than those who did not undergo education (*M* = 4.52). The interaction between the two factors was not supported in the original study, *p* > .950.

Thus, though we also find no interaction effects, we fail to replicate the main effects of opportunity cost and education.

#### Forced choice (extension)

To analyze the two-alternative forced choice responses, we built a generalized linear model (GLM). We have already built this model for our later exploratory analyses (see section ‘Study 1 versus Study 5: Analysis of within-subject effects’ for details of the model building procedure) which included the same two independent variables as the ANOVA, namely opportunity cost, education, and their interaction, as well as an additional independent variable and other interactions. In that generalized linear model, we coded the factors such that we get the results for the current study, therefore we report the results for the two main effects of interest and their interaction.

Specifically, the GLM showed no support for a main effect of opportunity cost (OR = .97 [.38, 2.46], *p* = .951), no support for a main effect of education (OR = 1.21 [.64, 2.30], *p* = .552), and no support for an interaction (OR = 2.00 [.56, 7.23], *p* = .285) – see Figure 1C right-hand side as well as Table 5.

**Table 5:** Results of linear (DV: Preference) and generalized linear (DV: Binary choice) models from the additional within-subjects analysis.


PREDICTORS	PREFERENCE			TWO-ALTERNATIVE FORCED CHOICE		
	
	*ESTIMATE*	*95% CI*	*p*	*ODDS RATIO*	*95% CI*	*p*

Opportunity cost	.09	–.46, .63	.758	.97	.38, 2.46	.951

Study	.15	–.23, .54	.437	1.37	.75, 2.57	.311

Education	.22	–.17, .60	.273	1.21	.64, 2.30	.552

Opportunity cost × study	–.46	–1.23, .31	.240	.65	.19, 2.23	.497

Opportunity cost × education	.48	–.30, 1.25	.228	2.00	.56, 7.23	.285

Study × education	.08	–.47, .63	.770	1.04	.45, 2.43	.922

Opportunity cost × study × education	.31	–.79, 1.41	.578	1.67	.31, 9.11	.551


### Discussion

Not in line with the results report in the target article, we failed to find evidence in support of facilitation of accounting for time, either using education or in highlighting opportunity costs. This suggests no support for H2a.

## Summary of results

### Replication results

We summarized our findings in Table 1.

In our replication of Soman’s (2001) Study 1, we found support for sunk costs in money larger than sunk costs for time (H1), although with weaker effects. Our extension using a continuous preference variable supported the conclusions of the replication.

In our replication of Study 2, we found mixed evidence relative to the original. On the one hand, our findings are consistent with Soman’s (2001) when comparing the sunk cost with the no sunk cost conditions in the money domain. On the other hand, we also found support for an effect in the time domain, inconsistent with Soman’s (2001) findings. Moreover, we found that the effect in the time domain was larger than in the money domain.

In our replication of Study 5, we failed to find any support for any of the effects, inconsistent with two main effects of education and opportunity cost in Soman’s (2001). We found no indication of benefits from facilitation of accounting for time, not in line with H2a.

### Additional analyses and checks

#### Sunk cost effect stronger for money than for time: Re-analysis using logistic regression

To address H1, Soman (2001) conducted multiple chi-square tests. Specifically, in Study 2, he showed that in the money condition, the chi-square test found support for differences between sunk cost and no sunk cost conditions, whereas the same difference was not supported for the time condition.

A different way to approach H1 is to ask whether the likelihood of picking the option associated with sunk costs (rocket engine in Study 2) is different not only between levels of a single independent variable (sunk cost presence or sunk domain) but also whether there was an interaction between the two variables. To address this question, we conducted logistic regression analyses for Study 2 for both the original and the replication data as it allowed us to test the interaction effect.

We ran a logistic regression for Study 2. The dependent variable was coded as 0 (solar-powered pump) and 1 (rocket engine). The predictors in the model were sunk domain (money/time) and sunk cost presence (no sunk cost/sunk cost) as well as their interaction. We wanted our model to test whether there was a main effect of sunk domain in the sunk cost present condition (thereby replicating the effect from Study 1) and also whether there was a main effect of sunk presence, regardless of the sunk domain. In order to achieve this, we coded sunk domain as a sum contrast and sunk presence as a treatment contrast, with sunk cost present as its baseline condition. In order to get predicted probabilities from the model for main effects with no baseline condition for remaining factors (in this case the main effect for sunk presence), we applied marginal standardization, which has been reliably shown to be a robust method compared to alternatives (Muller & MacLehose, 2014; Williams, 2012).

**Figure 2:**
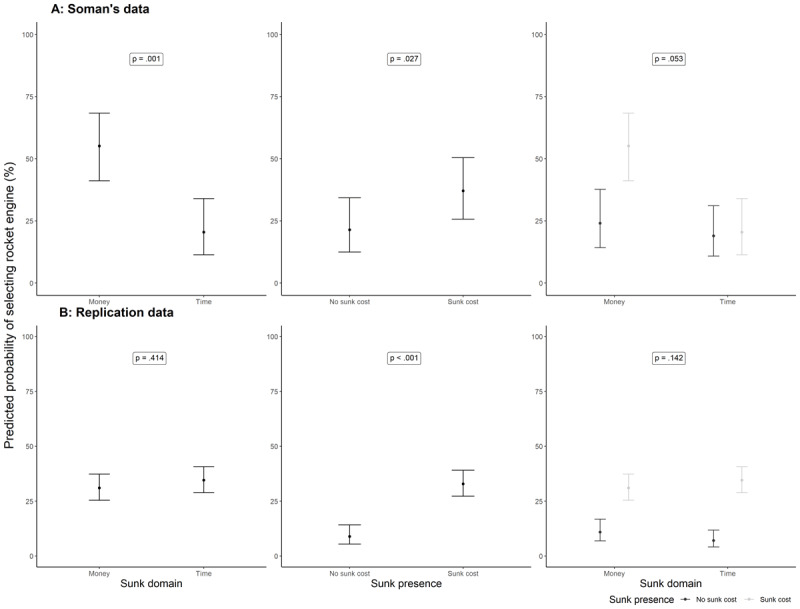
Study 2: Predicted probabilities from logistic regression analyses. *Note*. The main effect of sunk domain is plotted based on predicted probabilities from the sunk cost present condition, while the main effect of sunk presence is plotted using marginal standardization across levels of sunk domain.

The results of the logistic regression for Study 2 on Soman’s (2001) original data showed that there was support for a main effect of sunk domain, such that the odds of selecting the rocket engine design in the sunk cost condition went down by 79% in the domain of time compared to money (OR = .21 [.08, .50], *p* = .001). Soman’s data also revealed support for a main effect of sunk cost presence, regardless of sunk domain, such that the odds of selecting the rocket engine were 52% lower in the no sunk cost compared to the sunk cost condition (OR= .48 [.25, .92], *p* = .027; sunk cost effect differences between the money and time domains: OR = 3.55 [.99, 13.06], *p* = .053; see Figure 2A).

We conducted a logistic regression for Study 2 in our replication data and found a main effect of sunk presence (OR = .20 [.13, .30], *p* < .001), but no support for a main effect of sunk domain (OR = 1.17 [.80, 1.72], *p* = .414), and no support for an interaction (OR = .53 [.22, 1.23], *p* = .142) – see Figure 2B. See Table 6 for a summary.

**Table 6:** Summary of additional analyses.


HYPOTHESIS	QUESTION ADDRESSED	ANALYSIS USED	CONSISTENT WITH REPLICATION ANALYSIS (YES/MIXED/NO)	DETAILS

The sunk cost effect is weaker for time than for money.	Does the likelihood of picking the option associated with sunk costs (rocket engine in Study 2) vary significantly between levels of one independent variables (sunk cost presence or sunk domain) given a change in the other (i.e., an interaction effect)?	2x2 logistic regression on both Soman’s original data as well as the replication data.	Yes	Both re-analyses and replication analyses are not in-line with the hypothesis: the replication analyses showed comparable to larger effect size for time than for money, whereas the reanalyses show no support for domain differences.

Facilitation of moneylike accounting by using education about economic approaches to time strengthens the sunk cost effect of time (tested only in the time domain).	What are the differences between Study 1 and the high versus low opportunity cost conditions in Study 5 (i.e., study by opportunity cost interaction, in the no education condition in Study 5)?	Two linear models: one linear model with preference ratings as the dependent variable and one generalized LM with 2-alternative ticket choice as the dependent variable. The models included three independent variables: study (Study 1 vs Study 5), opportunity cost (low vs high), education (no education vs education), and all their interactions.	Yes	Both no support for interactions show that, at least in the time domain, neither the opportunity cost, nor the education manipulations, made a difference. Although this is aligned with the replication analyses in our sample, it is not in-line with Soman’s (2001) conclusion.
	
	Are differences between Study 1 and the high versus low opportunity cost conditions in Study 5 affected by education (study by opportunity cost by education interaction)?		Yes	


#### Study 1 versus Study 5: Analysis of within-subject effects

We extended the original analyses of H2 by considering an additional within-subject factor: study. Specifically, we took advantage of three of our design choices: 1) the replications of Study 1 and Study 5 both involved the same theatre performance versus rock concert ticket scenario, with the only difference that the design of Study 5 was a 2 × 2 between-subjects; 2) the same participants completed both Study 1 and Study 5 in the same survey; 3) we included both the two-alternative forced choice and the Likert response scales in both Study 1 and Study 5.

This allowed us to address two additional questions: 1) What are the differences between Study 1 and the high versus low opportunity cost conditions in Study 5 (i.e., study by opportunity cost interaction, in the no education condition in Study 5), and 2) Are those differences affected by education (study by opportunity cost by education interaction). To test both questions, we focused on the time sunk cost domain, as Study 5 only included the vignette version in the time domain.

To address these questions, we constructed two linear models: one linear model (LM) with preference ratings on a continuous scale as the dependent variable and one generalized LM (GLM) with ticket choice (theatre performance coded as 1 and rock concert as 0) as the dependent variable. We included three independent variables: study (Study 1 vs. Study 5), opportunity cost (low vs. high), education (no education vs. education), and all their interactions. The factor variables were coded such that study was set as a treatment contrast, with Study 5 as the baseline condition, opportunity cost was coded as a sum contrast, and education was coded as a treatment contrast, with no education as the baseline condition.

The results of both models showed no two-way interaction between study and opportunity cost in the no education condition, and no three-way interaction between opportunity cost, study and education in either of the models—see Table 5 for outputs of those models and Table 6 for a summary of the results. Our findings for Study 5 indicate that no support for the introduction of opportunity cost, with or without education, as having any impact relative to Study 1.

#### Order effects between studies

One deviation from the original study is that all participants completed all scenarios. We considered this to be a stronger design with many advantages that we laid out in the ‘Studies overview’ section above, yet one disadvantage is that answers to one scenario may bias participants’ answers to following scenarios (recall that Study 1 and 2 were presented in random order followed by Study 5). To address this is to run all analyses for each of the studies by only focusing on the participants that completed that study first. We found no differences in conclusions—see Table 7.

**Table 7:** All analyses re-run, split by whether Study 1 or Study 2 was presented first.


ANALYSIS	STATISTICAL TEST AND FACTORS		FULL SAMPLE *N* = 821		STUDY 1 FIRST *N* = 393		STUDY 2 FIRST *N* = 428	
		
			ES	*p*	ES	*p*	ES	*p*

Replication analyses								

Study 1: Forced choice	Chi-square		.38	< .001	.41	< .001	.36	< .001

Study 1: Preference	Independent samples t-test		–.79	< .001	–.74	< .001	–.84	< .001

Study 2: Time domain	Chi-square		.32	< .001	.36	< .001	.29	< .001

Study 2: Money domain	Chi-square		.23	< .001	.28	< .001	.19	< .001

Study 5: Preference	between-groups ANOVA	opportunity cost	.00	.284	.01	.118	.00	.969

		education	.00	.150	.01	.058	.00	.850

		opportunity cost × education	.00	.308	.00	.359	.00	.527

Study 5: Forced choice	Generalized Linear Model	opportunity cost	.97	.951	1.40	.619	.68	.570

		education	1.21	.552	1.75	.207	.82	.693

		opportunity cost × education	2.00	.285	1.22	.825	3.69	.185

Additional analyses and checks								

Study 2 re-analysis	Logistic Regression	sunk domain	1.17	.414	1.01	.960	1.35	.282

		sunk presence	.20	< .001	.15	< .001	.26	< .001

		sunk type × sunk presence	.53	.142	.49	.291	.55	.297

Study 1 versus Study 5: Analysis of within subject effects	Linear model	opportunity cost	.09	.758	.35	.370	–.23	.566

		study	.15	.437	.03	.901	.29	.308

		education	.22	.273	.44	.118	.05	.852

		opportunity cost × study	–.46	.240	–.38	.486	–.54	.342

		opportunity cost × education	.48	.228	.42	.457	.65	.234

		study × education	.08	.770	.33	.412	–.18	.647

		opportunity cost × study × education	.31	.578	–.01	.986	.62	.424

	Generalized Linear Model	opportunity cost	.97	.951	1.40	.619	.68	.570

		study	1.37	.311	1.23	.650	1.43	.436

		education	1.21	.552	1.75	.207	.82	.693

		opportunity cost × study	.65	.497	.93	.939	.49	.436

		opportunity cost × education	2.00	.285	1.22	.825	3.69	.185

		study × education	1.04	.922	1.24	.722	.94	.928

		opportunity cost × study × education	1.67	.551	1.29	.832	2.02	.597


*Note*. Reported effect sizes (ES) are: Chi-square – ϕ_c_, Independent samples t-test – Cohen’s *d*, ANOVA –, Generalized Linear Model and Logistic Regression – Odds Ratios, Linear model – β.

#### Exploratory comprehension questions analyses

**Figure 3:**
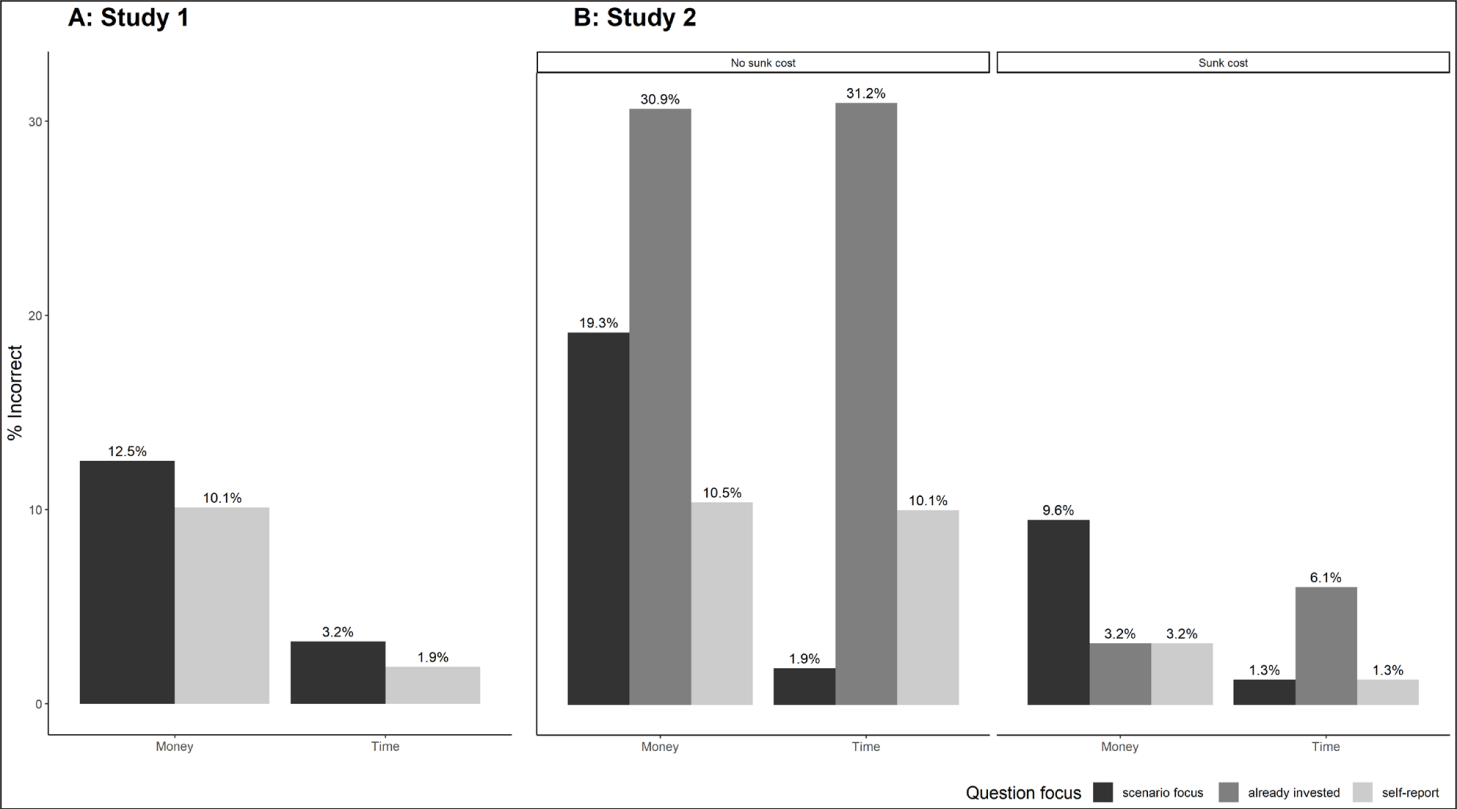
Percent of incorrect responses on comprehension questions across the entire sample.

Given the large number of participants excluded due to failing a comprehension question (70% of all excluded), we decided to investigate further. There were two questions in Study 1 and 3 questions in Study 2. The first question was about the focus of the scenario which asked, ‘What was the cost of the tickets, time or money?’ in Study 1 and ‘What was the scenario mainly focused on, time or money?’ in Study 2. The second question was a self-report on understanding which was phrased ‘Did you correctly understand the scenario the first time…?’ for both studies. The third question appeared only in Study 2 and was about whether the participant correctly understood if they have already invested something: ‘Have you already invested anything in your current design?’ The order of these questions was such that the scenario focus and the already invested questions (the latter for Study 2 only) appeared on the same page then on the next page was the comprehension quiz participants had to take and on the following page was the self-report question. The percentage of people who responded incorrectly out of everyone who completed the question (before exclusions) is found in Figure 3.

Further exploratory analyses revealed two notable trends. First, across both studies and all questions, participants failed a comprehension question more often in the money condition compared to the time condition, χ^2^(1) = 65.71, *p* < .001, ϕ_c_ = .10, 95% CI [.08, .13]. Second, in Study 2 across all questions, participants failed a comprehension question in the no sunk cost condition more often than in the sunk cost condition, χ^2^(1) = 170.05, *p* < .001, ϕ_c_ = .21, 95% CI [.18, .25].

These findings suggest two observations. First, given the high rate at which participants reported some lack of comprehension, we think our study was well-designed to incentivize people to report their understanding truthfully, without limiting their pay and incentivizing them to lie. Second, we believe that the presence (or lack thereof) of comprehension questions in hypothetical scenarios, often used in studies of the sunk cost effect, can have major impact on the data quality, and so we encourage further research to add them to gain further insights into potential comprehension issues.

We therefore believe that it was important to include exploratory comprehension questions after the target’s replication scenarios, and that our findings are likely robust given that we ensured that the participants included in the final sample read and understood the scenarios as intended.

## General Discussion

We conducted a close, high-powered, replication and extension Registered Report of Soman’s (2001) Studies 1, 2, and 5 and found mixed results.

In line with Soman’s (2001) findings, we found that, based on Study 1, participants show a stronger sunk cost effect in money than in time. However, in our replication of Study 2, we found evidence for both sunk money costs and sunk time costs. In our replication of Study 5, we found no evidence suggesting that manipulating either the strength of the opportunity costs or educating people about economic approaches to time had any impact on sunk time cost effect.

Similar to previous studies, we found strong evidence for sunk money costs (Arkes & Blumer, 1985; Bornstein et al., 1999; Coleman, 2009; Navarro & Fantino, 2005; Soman & Cheema, 2001). Our findings also seem to be in line with some of the existing literature suggesting sunk time costs seem volatile, depending on context.

One possible reason for this could be that whereas Soman (2001) recruited undergraduate students across his studies, our sample was from the general population. These groups could be different based on age, which differentially affects susceptibility to sunk costs (Strough et al., 2008), based on the experience they have accounting for time (Bornstein et al., 1999; DeVoe & Pfeffer, 2007; Ronayne et al., 2021), and based on cultural influences, which might make our sample more sensitive to accounting for time, given the greater emphasis placed productivity and time-management in modern discourse. We also note that conducted our study right after the world started coming back to normal after the COVID-19 pandemic, which may have impacted people’s appreciation of time.

Although differences abound between our replication and the target’s, the inconsistent mixed results of our replications of Studies 1 and 2 in relation to the strength of the sunk time cost effect are more difficult to explain. One of the major strengths of our replication was that all participants completed all studies, thus rendering explanations that refer to between-sample differences unlikely. Still, we find that the sunk money cost effect is stronger than the sunk time cost effect in Study 1, but with no indication for differences between the two in Study 2. This difference could not be explained by the randomized order of the studies, the exclusion criteria, or the comprehension of the scenarios. This likely suggests that there are other factors that further complicate the study of sunk cost effects that would need to be addressed by future research.

### Limitations of our replication and directions for future research

Our replication had limitations that can be broadly split into two categories: contextual differences and implementation.

The context surrounding how people think about time and money sunk costs might have changed over time. Our replication was conducted more than 20 years after the original thus how people approach time and money may have changed. This is partly why ongoing repeating replications are needed, to keep our knowledge about an important phenomenon up to date. In addition to repeating replications, we call for regular systematic reviews and/or meta-analyses that would help summarize the growing literature, address and help explain seemingly discrepant results (such as our differences in patterns of results across Studies 1 and 2), and would drive the development of better theories and empirical investigations. Another factor that could influence how people think about time and money sunk costs was sample differences: we used a sample from the general population, which might systematically differ on demographic characteristics that are correlated with the sunk cost effect compared to the student sample used in the original. For instance, our replication sample was likely twice as old as that of the original, which might make them more sensitive to any further time investments (e.g., Strough et al., 2008), given that sunk cost calculations are done relative to one’s total available resources, rather than taken as absolute (Garland & Newport, 1991). Following similar logic, it is possible that the social class of the different samples could also have an impact. The influence of such demographic factors is speculative at best and further research is needed to disentangle these open questions.

The implementation of our replication also introduced some limitations, especially in our exploratory conceptual replication of Study 5. First, we made adjustments to the opportunity cost manipulation. Second, in the original, the education intervention was implemented by manipulating when the study was conducted—either before a classroom discussion about the economic value of time (control condition) or after (education condition)—whereas in our replication, the intervention was implemented by having participants read information on the screen and complete comprehension checks. These changes were necessary given the change in the medium, yet it may have affected the results. Third, the studies were originally run separately, and in our design, we ran the studies together, with Study 5 always last, given its similarity to Study 1. This allowed us to gain additional insights and conduct a comparison between Study 1 and Study 5, yet this does mean that our adjustments make the replication of Study 5 less direct in comparison to Studies 1 and 2, with higher likelihood of the results being different than that of the target.

## Conclusion

We conducted a Registered Report of a close, high-powered, replication and extension of Studies 1 and 2 and a conceptual replication of Study 5 in Soman (2001), testing the predictions that sunk cost effect is weaker in the time domain than in the money domain, and that the facilitation of money-like accounting for sunk time costs would strengthen the sunk time cost effect. We concluded mixed support: a) sunk time cost effect was stronger than the sunk money cost in Study 1, yet no support for differences between the two in Study 2, and b) we found no indication of facilitation of money-like accounting as having any impact on the sunk time cost effect. We tested and ruled out several study order or exclusions as the possible explanations and discussed directions for future research.

See recommendation and open peer-review on: https://rr.peercommunityin.org/articles/rec?id=452.

## Additional File

The additional file for this article can be found as follows:

10.5334/irsp.883.s1 Supplementary Materials.

## Contributor Roles Taxonomy

**Table:** 


ROLE	NIKOLAY PETROV	YIN KAN CHAN, CHEUK NAM LAU, TIN HO KWOK. LOK CHING CHOW, WAI YAN LO	WENKAI SONG	GILAD FELDMAN

Conceptualization		X		X

Data curation	X	X		X

Formal analysis	X	X		

Funding acquisition				X

Investigation	X	X		

Preregistration verification	X		X	

Data analysis verification	X			

Methodology	X	X		

Project administration				X

Resources	X	X		X

Software	X	X		

Supervision				X

Validation	X			

Visualization	X	X		

Writing – original draft	X	X		

Writing – review and editing	X		X	X


## Footnotes

The ~40% exclusion rate we find is significantly higher than the 15% we anticipated in our preregistration. 70% (373 of 527) of those that were excluded were due to failing a comprehension question. We further investigate this in the “Comprehension question analysis” subsection below.

For the money condition, we adjusted the original’s absolute values to account for inflation and meet updated minimum wages in the US. Specifically, the original article, published in 2001, promised compensation to the hypothetical student in an imagined scenario equivalent to US$20 for 5 hours of work and US$60 for 15 hours of work. We adjusted these numbers to US$75 and US$225 for 5 and 15 hours of work, respectively.

The original did not report standard deviations.
