# Supplementary material for: Sunk Cost Effects for Time Versus Money: Replication and Extensions Registered Report of Soman ()
Source: Int Rev Soc Psychol. 2023 Nov 28;36:17. doi: 10.5334/irsp.883 (PMC12372683; doi:10.5334/irsp.883)
Supplement: Supplementary file 1 — Supplementary Materials. [file irsp-36-883-s1.pdf]

Sunk cost effects for time versus money:
Replication and extensions Registered Report of Soman (2001)

Supplementary materials

Table of Contents

Effect sizes calculation 2
Comparisons and deviations 3
  Replication classification 3
  Pre-exclusion vs post-exclusion results comparison 4
Open science disclosures 5
Materials used 6
  Study 1 6
    Manipulation 6
    Measures 7
    Comprehension checks 7
    Familiarity check 9
  Study 2 10
    Manipulation 10
    Measures 11
    Comprehension checks 11
    Familiarity check 13
  Study 5 14
    Manipulation: Opportunity cost education 14
    Comprehension check questions 14
    Manipulation: Opportunity cost 16
    Measures 17
  Funneling section 17
Additional information about the study 18
References 19

Effect sizes calculation

In order to calculate the φ_c effect size of the Soman's (2001) Studies 1 and 2, where a chi-squared test was used, we inputted the raw frequencies into R and used the package *DescTools* (v0.99.44) and the function *CramerV* to calculate the φ_c statistic and a 95% confidence interval. The same function was used for the replication.

In order to calculate the effect sizes of the ANOVA test for the original, we used R's package *effectsize*, in order to calculate η_p^2 (function: *F_to_eta2*). We used the R package *MOTE* to calculate the ω^2 (function: *omega.F*). In order to calculate both the η_p^2 and the ω^2 for the replication, we used the package *sjstats* (function: *anova_stats)*. In order to obtain Cohen's *f* effect size measure for the power analyses, the package *effectsize* was used (function: *F_to_f*), which translated an F statistic to Cohen's *f*.

These calculations can be found in the R script in the online repository on OSF, files “Soman 2001 - Power analysis script.Rmd” and “Soman 2001 - Power analysis script.html”.

Comparisons and deviations

Replication classification

We summarized our replications as very close for Studies 1 and 2 and between close and far replication for Study 5, using the classification criteria set out by LeBel et al. (2018).

Design facet	Study 1	Study 2	Study 5
Effect, Hypothesis	Same	Same	Same
IV Construct	Same	Same	Same
DV Construct	Same	Same	Same
IV Operationalization	Same	Same	Same
DV Operationalization	Same	Same	Same
Population (e.g., age)	Similar	Similar	Similar
IV Stimuli	Same	Same	Different
DV Stimuli	Same	Same	Different
Procedural Details	Similar	Similar	Different
Physical Setting	Different	Different	Different
Contextual Variables	Different	Different	Different
**Replication classification**	Very close replication	Very close replication	Close/far replication

Pre-exclusion vs post-exclusion results comparison

Exclusion criterion	N excluded
Did not agree to pay attention/participate	33
Non-native	28
Dropped out	68
Failed scenario comprehension	373
Low proficiency in English and understanding of materials (<5 on a 1-7 scale)	2
Low seriousness (<4 on 1-5 scale)	1
Familiarity with the materials	22

	Test	Exclusions	Test statistic	*df*	*p*	Effect size	Difference
Study 1	Chi-square; difference between sunk time and sunk money conditions in rate of choosing a ticket	Before	χ^2 = 140.02	1	< .001	φ_c = .34	No difference
		After	χ^2 = 120.12	1	< .001	φ_c = .38	
Study 2	Chi-square; difference between sunk time and no sunk time conditions in rate of choosing a project	Before	χ^2 = 48.57	1	< .001	φ_c = .28	No difference
		After	χ^2 = 45.28	1	< .001	φ_c = .32	
	Chi-square; difference between sunk money and no sunk money conditions in rate of choosing a project	Before	χ^2 = 26.86	1	< .001	φ_c = .21	No difference
		After	χ^2 = 21.40	1	< .001	φ_c = .23	
Study 5	ANOVA; opportunity cost main effect	Before	*F* = 3.71	1, 817	.054	η_p^2 = .00	No difference
		After	*F* = 1.15	1, 817	.284	η_p^2 = .00	
	ANOVA; education main effect	Before	*F* = 6.09	1, 817	.014	η_p^2 = .00	No difference
		After	*F* = 2.08	1, 817	.150	η_p^2 = .00	
	ANOVA; opportunity cost by education interaction	Before	*F* = .29	1, 817	.592	η_p^2 = .00	No difference
		After	*F* = 1.04	1, 817	.308	η_p^2 = .00	

Open science disclosures

Item	***Disclosure***
Materials, data, and analysis scripts	Materials, data (raw and cleaned, with identifiers removed), and analysis scripts have been made publicly available on the Open Science Framework https://osf.io/pm264/.
Data collection	Data collection was completed before any analysis.
Conditions reporting	All collected conditions have been reported.
Data exclusions	Details have been reported in this document.
Variables reporting	All variables collected for this study have been reported and included in the provided data.

Materials used

Study 1

Manipulation

Participants were randomized into either a sunk time or a sunk money condition and read a hypothetical scenario.

**Sunk time scenario**	**Sunk money scenario**
**Theater performance or rock concert #1**<br><br>Imagine you are a student and you recently saw an advertisement on the student bulletin board. A literature professor was looking for a research assistant to work for about 15 hours. The payment was in the form of a front row seat to a professional theater performance.<br><br>On the same bulletin board, a music professor was also looking for a research assistant to work for about five hours, and this assistant would be paid with a ticket (in a good section) to a rock concert by a band that you like.<br><br>You had recently seen posters for both the theater performance and the rock concert. You think you will like to see both these events, although you expect to like the rock concert more.<br><br>You work for both the professors - 15 hours for literature and 5 hours for music - and get paid with the two tickets (theater and rock concert respectively).<br><br>As you are putting the tickets away in your wallet, you notice that both events are scheduled for the same evening and are both at good locations on campus. The tickets are non-transferable. nor can they be exchanged. You can use only one of the tickets and not the other.	**Theater performance or rock concert #1**<br><br>Imagine you are a student and you recently saw an advertisement on the student bulletin board. A literature professor was looking for a research assistant to work for about 15 hours. The total payment for this job will be US$225.<br><br>On the same bulletin board, a music professor was also looking for a research assistant to work for about five hours, and this assistant would be paid a total of US$75.<br><br>You had recently seen posters for a professional theater performance as well as a rock concert by a band that you like. You think you would like to see both these events, although you expect to like the rock concert more.<br><br>You work for both the professors - 15 hours for literature and 5 hours for music - and get paid US$225 for the 15 hours literature work, and US$75 for the 5 hours music work, a total of US$300. Given that you had the money from these jobs, you purchased tickets for both these events. The ticket for the theater cost US$225 while a ticket to the rock concert cost US$75.<br><br>As you are putting the tickets away in your wallet, you notice that both events are scheduled for the same evening and are both at good locations on campus. The tickets are not transferable, nor can they be exchanged. You can only use one of the tickets and not the other

Measures

Forced choice (replication)

In both condition the participants were asked:

Which ticket will you use?

- o Theater performance
- o Rock concert

Preference (extension)

Which ticket will you use? Please indicate your preference for attending the rock concert vs. theater on the scale below.

	Definitely Rock Concert				Indifferent				Definitely Theater Performance
	4	3	2	1	0	1	2	3	4
Which even do you prefer to attend?	O	O	O	O	O	O	O	O	O

These were presented to participants as 4 to 4 but coded as 1 to 9.

Comprehension checks

Right after the response, on a separate page, participants were asked:

Finally, in the scenario, to the best of your understanding:

What was the cost of the tickets, time or money?

- o Time (hours spent working)
- o Money (cash)

On the next page, participants were given a quiz, which they had to answer correctly in order to proceed to the next page. The page contained the entire scenario that they had previously seen as well as the following information and questions (same for both time and money conditions):

In this section we would like to check with you to verify your understanding of the previous scenario. The scenario below is the same as the one in the previous page, followed by comprehension questions.

<entire scenario that they had just seen>

To make sure that you read and understood the scenario, please answer the following comprehension questions.

How did you obtain the theater performance and rock concert tickets?

o I bought them myself with the money I had from the salary that I received from working for the literature professor (US$225) and the music professor (US$75).
o I received the tickets from the music and literature professors as compensation for the work I did for them

How did you get paid for the 15 hours work you did for the literature professor?

o I received US$225 salary, which I used to buy a theater performance ticket
o I received a theater performance ticket

How did you get paid for the 5 hours work you did for the music professor?

o I received US$75 salary, which I used to buy a rock concert ticket
o I received a rock concert ticket

Which event did you think you will like better?

o Theater performance
o Rock concert

After answering correctly all of the above comprehension quiz questions, participants were asked if their initial comprehension was accurate and what they would choose if they could choose again:

Now that you've answered the quiz and checked all answers, we want to check with you: Did you correctly understand the scenario the first you read it when you answered about your decision of which ticket to use?

Please note that the answer to this question will NOT in any way impact your current participation or your compensation. Please answer honestly to help us ensure accurate insights from this research.

o Yes, I understood the scenario correctly the first time when I made the decision which ticket to use
o No, the comprehension questions helped me realize that I did not understand the scenario correctly the first time when I made the decision which ticket to use.

Suppose you have the chance to choose again...

Which ticket will you use? If you had to choose which one to use, which would you use?

You can find the same scenario below if you need it to answer this question.

o Rock concert
o Theater performance

<entire scenario that they had just seen>

Familiarity check

After completing Study 1, participants were asked:

Have you ever been presented with a scenario similar to the one you have just read?

Please note that the answer to this question will NOT in any way impact your current participation or your compensation. Please answer truthfully.

- Answer scale:
    - Yes, I have seen this scenario before;
    - No, this is the first time I encounter this scenario.

If they responded positively, the were asked:

Where have you seen the scenario before?

- Answer scale: [single line text box]

Study 2

Manipulation

Participants were randomized to read one of four scenarios – 2 (*sunk cost domain*: time or money) x 2 (*sunk cost presence*: sunk cost or no sunk cost).

**Time**		**Money**	
**Sunk cost**	**No sunk cost**	**Sunk cost**	**No sunk cost**
**"New invention" competition**<br><br>Imagine you are a student and are planning to submit an entry to the 'new invention' competition organized by the students' club.<br><br>You have spent 30 hours preparing a design for an innovative rocket engine and estimate that it will take you an additional 10 hours to finish it.<br><br>You just learned that **the winner of the previous year's competition was also working on a rocket engine** design similar to yours.<br><br>You had also thought about working on an (equally innovative and good) design for a solar-powered pump that would take about 10 hours to complete.<br><br>You can submit only one entry, and since the deadline is very close, you must choose now. The question is - Should you spend 10 hours trying to finish your rocket engine design given what you know, or would you rather work on the solar-powered pump?	**"New invention" competition**<br><br>Imagine you are a student and are planning to submit an entry to the 'new invention' competition organized by the students" club.<br><br>You thought about preparing a design for an innovative rocket engine, and you estimate that it will take you 10 hours to finish it.<br><br>You just learned that **the winner of the previous year's competition was also working on a rocket engine** design similar to yours.<br><br>You had also thought about working on an (equally innovative and good) design for a solar-powered pump that would take about 10 hours to complete.<br><br>You can submit only one entry, and since the deadline is very close, you must choose now. The question is: Should you spend 10 hours trying to work on the rocket engine design or would you rather work on the solar-powered pump?	**"New invention" competition**<br><br>Imagine you are a student and are planning to submit an entry to the 'new invention' competition organized by the students' club.<br><br>You had already spent $90 on the rocket engine design. You expect that it will cost you an additional $30 to finish.<br><br>You just learned that **the winner of the previous year's competition was also working on a rocket engine** design similar to yours.<br><br>You had also thought about working on an (equally innovative and good) design for a solar-powered pump that would cost about $30 to complete.<br><br>You can submit only one entry, and since the deadline is very close, you must choose now. The question is: Should you spend $30 trying to finish your rocket engine design or would you rather work on the solar-powered pump?	**"New invention" competition**<br><br>Imagine you are a student and are planning to submit an entry to the 'new invention' competition organized by the students" club.<br><br>You thought about preparing a design for an innovative rocket engine, and you estimate that it will cost approximately $30 to finish it.<br><br>You just learned that **the winner of the previous year's competition was also working on a rocket engine** design similar to yours.<br><br>You had also thought about working on an (equally innovative and good) design for a solar-powered pump that would cost about $30 to complete.<br><br>You can submit only one entry, and since the deadline is very close, you must choose now. The question is: Should you spend $30 trying to work on the rocket engine design or would you rather work on the solar-powered pump?

Measures

In all cases **participants had two options**:

- Rocket engine
- Solar-powered pump

Comprehension checks

Right after the response, on a separate page, participants were asked:

> Finally, in the scenario, to the best of your understanding:
>
> What was the scenario mainly focused on, time or money?
>
> - Time (hours spent working)
> - Money (cash)
>
> To the best of your understanding:
>
> Have you already invested anything in your current design?
>
> - Yes, I have already made some investment in the current design.
> - No, I have not yet invested anything in my design

On the next page, participants were given a quiz, which they had to answer correctly in order to proceed to the next page. The page contained the entire scenario that they had previously seen as well as the following information and questions (same for all conditions):

> In this section we would like to check with you to verify your understanding of the previous scenario. The scenario below is the same as the one in the previous page, followed by comprehension questions.
>
> <entire scenario that they had just seen>
>
> What did you spend so far in your work on the rocket engine?
>
> - Nothing
> - US$90
> - 30 hours
> - US$30
> - 90 hours
>
> What did you spend so far in your work on the solar powered pump?
>
> - Nothing
> - US$90
> - 30 hours
> - US$30
> - 90 hours
>
> What would it take you to finish the rocket engine design?
>
> - Nothing

- US$30
- 10 hours
- US$10
- 30 hours

What would it take you to finish the solar-powered pump design?

- Nothing
- US$30
- 10 hours
- US$10
- 30 hours

After answering correctly all of the above comprehension quiz questions, participants were asked if their initial comprehension was accurate and what they would choose if they could choose again:

Now that you've answered the quiz and checked all answers, we want to check with you: Did you correctly understand the scenario the first you read it when you answered about your decision of which ticket to use?

Please note that the answer to this question will NOT in any way impact your current participation or your compensation. Please answer honestly to help us ensure accurate insights from this research.

- Yes, I understood the scenario correctly the first time when I made the decision which ticket to use
- No, the comprehension questions helped me realize that I did not understand the scenario correctly the first time when I made the decision which ticket to use.

Suppose you have the chance to choose again...

Which ticket will you use? If you had to choose which one to use, which would you use?

You can find the same scenario below if you need it to answer this question.

- Rocket engine
- Solar-powered pump

<entire scenario that they had just seen>

Familiarity check

After completing Study 1, participants were asked:

Have you ever been presented with a scenario similar to the one you have just read?

Please note that the answer to this question will NOT in any way impact your current participation or your compensation. Please answer truthfully.

- Answer scale:
    - Yes, I have seen this scenario before;
    - No, this is the first time I encounter this scenario.

If they responded positively, the were asked:

Where have you seen the scenario before?

- Answer scale: [single line text box]

Study 5

Manipulation: Opportunity cost education

Participants were first randomized to either receive opportunity cost education or not. If they did, they saw the following information:

<u>Importance of considering time as opportunity cost</u>

NB: You will be asked short questions to ensure you have understood this information.

Below is a short passage on opportunity cost which is relevant for the kinds of decisions that you answered. Please read it carefully. We will present you with a similar decision to the ones you already made once you finished reading:

<u>Opportunity cost refers to the highest-valued option forgone in making a choice</u>. It includes both monetary costs (i.e. highest-valued alternative use of the money) and time costs (i.e. highest-valued alternative use of the time).

<u>We often overlook the more implicit time costs when considering opportunity costs</u>.

Let us look at an example: You are going to take a 3-day stay-cation in a hotel next to a glamorous lake. The expenditures during the whole stay-cation, such as food expenses, will cost you $50. You have booked the hotel for the stay-cation at $250 and the price is non-refundable. In those 3 days, you could have worked for a temporary job in a book fair, where you could earn $600. What is your total opportunity cost of going on the stay-cation?

In this problem, the $50 food expenditure is a part of the opportunity cost as you directly incur a payment due to the stay-cation plan. However, since the $250 hotel fee has been paid and is non-refundable, you could not retrieve the amount whether you decide to join the stay-cation or not. Hence, the hotel fee is not part of the opportunity cost. Lastly, the $600 salary is said to be a time cost since you forgo the option to work at the book fair if you choose to go on the stay-cation. Thus, the opportunity of earning income is forgone and it is counted as an implicit cost.

Therefore, the opportunity cost in this scenario is $(50+600) = $650.

This example illustrates why **<u>time cost is a part of the opportunity cost in an economic sense</u>**.

Comprehension check questions

If participants read this information they were then asked two instructional comprehension check questions which they had to answer correctly to proceed:

What does opportunity cost refer to?

- The cost you incur when you engage in any activity.

- The cost incurred by not engaging in an alternative activity of higher value relative to the chosen activity.
- The cost associated with an activity that has both benefits and downsides.

Why was $600 part of the opportunity cost calculation in the scenario you read?

- It was the salary from a temporary job.
- It was a high value option.
- It was the time cost associated with forgoing to work at the book fair at the expense of going to the stay-cation.

Manipulation: Opportunity cost

Afterwards, participants read one of two scenarios, depending on whether they were in the high or low opportunity cost condition:

**High opportunity cost**	**Low opportunity cost**
**Theater performance or rock concert #2 [Revisited]**<br><br>(IMPORTANT: we added new information highlighted in the large text below, please reread the paragraph and consider the new information)<br><br>Imagine you are a student and you recently saw an advertisement on the student bulletin board. A literature professor was looking for a research assistant to work for about 15 hours. The payment was in the form of a front row seat to a professional theater performance.<br><br>On the same bulletin board, a music professor was also looking for a research assistant to work for about five hours, and this assistant would be paid with a ticket (in a good section) to a rock concert by a band that you like.<br><br>You had recently seen posters for both the theater performance and the rock concert. You think you will like to see both these events, **although you expect to like the rock concert more.**<br><br>It is the fall semester - you were taking five classes and working three part-time jobs to support yourself. As a result, **you have been badly pressed for time**. Yet, you work for both the professors - 15 hours for literature and 5 hours for music - and get paid with the two tickets (theater and rock concert respectively).<br><br>As you are putting the tickets away in your wallet, you notice that both events are scheduled for the same evening and are both at good locations on campus. The tickets are non-transferable. nor can they be exchanged. You can use only one of the tickets and not the other.	**Theater performance or rock concert #2 [Revisited]**<br><br>(IMPORTANT: we added new information highlighted in the large text below, please reread the paragraph and consider the new information)<br><br>Imagine you are a student and you recently saw an advertisement on the student bulletin board. A literature professor was looking for a research assistant to work for about 15 hours. The payment was in the form of a front row seat to a professional theater performance.<br><br>On the same bulletin board, a music professor was also looking for a research assistant to work for about five hours, and this assistant would be paid with a ticket (in a good section) to a rock concert by a band that you like.<br><br>You had recently seen posters for both the theater performance and the rock concert. You think you will like to see both these events, **although you expect to like the rock concert more.**<br><br>It is the summer - you did not have to take any classes and did not have to work to support yourself. Therefore, **there is relative flexibility in your schedule.** You work for both the professors - 15 hours for literature and 5 hours for music - and get paid with the two tickets (theater and rock concert respectively).<br><br>As you are putting the tickets away in your wallet, you notice that both events are scheduled for the same evening and are both at good locations on campus. The tickets are non-transferable. nor can they be exchanged. You can use only one of the tickets and not the other.

Measures

In both conditions participants were asked:

Preference (extension)

Which ticket will you use? Please indicate your preference for attending the rock concert vs. theater on the scale below.

	Definitely Rock Concert				Indifferent				Definitely Theater Performance
	4	3	2	1	0	1	2	3	4
Which even do you prefer to attend?	o	o	o	o	o	o	o	o	o

These were presented to participants as 4 to 4 but coded as 1 to 9.

[Note: this is a deviation from the original's 1 to 9 scale]

Forced choice(extension)

Which ticket will you use?

- Theater performance
- Rock concert

Funneling section

Three funneling questions:

- How serious were you in filling out this questionnaire?
  - Answer scale: 1 (not at all) to 5 (very much)
- Have you ever seen the materials used in this study or similar before?
  If yes - please indicate where.
  - Answer scale: No; Yes (if yes, please write in the box below where)
- What do you think the purpose of the study was? (one sentence)
  - Answer scale: [free text box]
- Help us improve for the next studies - Did you spot any errors? Anything missing or wrong? Something we should pay attention to in next runs? (briefly)
  - Answer scale: [free text box]

Additional information about the study

1. Setting: The study was conducted online via an online questionnaire using Qualtrics. There was no fixed physical setting in which the study was conducted. In addition, we did not disallow participation using any specific devices.
2. Duration of Study Sessions: Participants were allowed 30 minutes to complete all study materials, sessions ended earlier if participants completed study earlier. Participants completed the questionnaire for an average of 12.23 minutes.
3. Time of Day: As questionnaires are conducted online, there is no limit to what time of the day the participants should complete the questionnaire. They could do it at any time of their convenience.
4. Data collection dates: Data collection started and ended on 12/March/2023.
5. Participant Recruitment: Participants were recruited using Amazon Mechanical Turk using CloudResearch.

Data collection procedures

This study was conducted on Amazon Mechanical Turk with American participants. We imposed the following settings in recruiting our participants:

1. Participants were paid $1.75 as a fixed participation reward. This amount was determined by multiplying the expected completion time (in mins.) with the minimal federal wage in the U.S. (i.e., $0.121 per minute).
2. The expected completion time was set at 12 minutes in advance.
3. The most time we allowed each worker to complete the study was 30 minutes.
4. We limited all workers' HIT Approval Rate to be between 95% and 100%.
5. We limited each worker's number of HITs approved to be between 5,000 and 500,000.
6. We blocked Suspicious Geocode Locations and Universal Exclude List Workers.
7. We blocked duplicate IP addresses and duplicate geolocation.
8. We enabled HyperBatch so that all eligible workers were able to participate in our HIT immediately after the survey was launched.
9. We restricted workers' location to be in the U.S.

References

LeBel, E. P., McCarthy, R. J., Earp, B. D., Elson, M., & Vanpaemel, W. (2018). A Unified Framework to Quantify the Credibility of Scientific Findings. *Advances in Methods and Practices in Psychological Science*, *1*(3), 389–402. https://doi.org/10.1177/2515245918787489
